# Pharmacokinetic Properties of 2^nd^-Generation Fibroblast Growth Factor-1 Mutants for Therapeutic Application

**DOI:** 10.1371/journal.pone.0048210

**Published:** 2012-11-01

**Authors:** Xue Xia, Joseph P. Babcock, Sachiko I. Blaber, Kathleen M. Harper, Michael Blaber

**Affiliations:** 1 Department of Biomedical Sciences, Florida State University, Tallahassee, Florida, United States of America; 2 Biomedical Research Laboratory Animal Resources, Florida State University, Tallahassee, Florida, United States of America; University of Iowa, United States of America

## Abstract

Fibroblast growth factor-1 (FGF-1) is an angiogenic factor with therapeutic potential for the treatment of ischemic disease. FGF-1 has low intrinsic thermostability and is characteristically formulated with heparin as a stabilizing agent. Heparin, however, adds a number of undesirable properties that negatively impact safety and cost. Mutations that increase the thermostability of FGF-1 may obviate the need for heparin in formulation and may prove to be useful “2nd-generation” forms for therapeutic use. We report a pharmacokinetic (PK) study in rabbits of human FGF-1 in the presence and absence of heparin, as well as three mutant forms having differential effects upon thermostability, buried reactive thiols, and heparin affinity. The results support the hypothesis that heparan sulfate proteoglycan (HSPG) in the vasculature of liver, kidney and spleen serves as the principle peripheral compartment in the distribution kinetics. The addition of heparin to FGF-1 is shown to increase endocrine-like properties of distribution. Mutant forms of FGF-1 that enhance thermostability or eliminate buried reactive thiols demonstrate a shorter distribution half-life, a longer elimination half-life, and a longer mean residence time (MRT) in comparison to wild-type FGF-1. The results show how such mutations can produce useful 2nd-generation forms with tailored PK profiles for specific therapeutic application.

## Introduction

A number of major diseases have an insufficiency in blood flow (ischemia) as a primary contributing pathology. Coronary ischemia, peripheral artery disease, and chronic non-healing wounds in diabetic ulcers and bed sores, for example, are fundamentally ischemic diseases. Specific cell types are associated with the growth of new blood vessels and wound healing, including endothelial cells, fibroblasts, and keratinocytes. Almost 50 years ago, normal human proteins capable of causing the proliferation of specific cell types were identified and termed “growth factors” [1]–[4]. More recent pre-clinical and clinical studies have demonstrated beneficial effects of the application of such growth factors in the treatment of ischemic disease; studies have reported the effective growth of new blood vessels in cardiac muscle of “no-option” heart patients after injection of a pro-angiogenic growth factor at the site of ischemia [5]–[7], as well as substantial acceleration of wound healing with topical application of pro-angiogenic growth factors at the site of full-thickness dermal injury and diabetic ulcers [8]–[12].

Several different growth factors have been evaluated in such “pro-angiogenic therapy” including vascular endothelial cell growth factor (VEGF) [13]–[15], FGF-1 [7], [9], [10], [16], FGF-2 [17], [18], platelet derived growth factor (PDGF) [19], [20] and keratinocyte growth factor (KGF) [21], [22]. Promising clinical results have been achieved, for example, using fibroblast growth factor-1 (FGF-1): FGF-1 is currently in NIH phase II clinical trials for the pro-angiogenic treatment of coronary heart disease (NCT00117936) and wound healing in diabetic foot ulcers (NCT00916292). FGF-1 is a relatively simple protein and can be expressed recombinantly using inexpensive bacterial fermentation. However, there are several issues that complicate the practical application of FGF-1 as a therapeutic agent. FGF-1 has an intrinsically poor thermostability (i.e., ΔG_unfolding_ = 21.1 kJ/mol [23]) and is prone to unfolding, aggregation, and subsequent loss of functional activity. Formulation studies to address the poor thermostability of FGF-1 have resulted in the common addition of heparin, which binds to, and stabilizes, FGF-1 [24], [25]. However, heparin is more expensive than FGF-1 to produce, is derived from pigs (with the potential for infectious agents), is highly heterogeneous, has its own (anti-coagulant) pharmacological properties, can initiate a serious allergic reaction, and can cause thrombocytopenia [26], [27]. Furthermore, while the treatment of coronary ischemia by FGF-1 has shown effectiveness with a single (injected) dose, healing of wounds is best achieved with multiple (topical) dosing – necessitating frequent removal of bandages for access to the wound area [8]–[10], [28]. This latter issue is complicated by the presence of heparin, which, due to its pharmacological properties, causes wounds to produce excessive exudate, diluting topically-applied therapeutics.

A potentially elegant solution to address negative aspects associated with heparin as a formulation additive for FGF-1 is to engineer the protein to increase the intrinsic thermostability. Such designed mutant proteins represent novel 2nd-generation forms. Over thirty different types of 2nd-generation recombinant human proteins with enhanced properties have been approved by the FDA for use as human pharmaceuticals [29]; examples include β-interferon (Betaseron®) and interleukin-2 (Proleukin®). A 2nd-generation form of FGF-1 that is stable in the absence of heparin would eliminate a number of undesirable consequences associated with this additive. Further gains in utility (especially for wound-healing application) could be realized if such mutants exhibit a PK profile providing extended *in vivo* elimination half-life or increased MRT.

**Figure 1:**
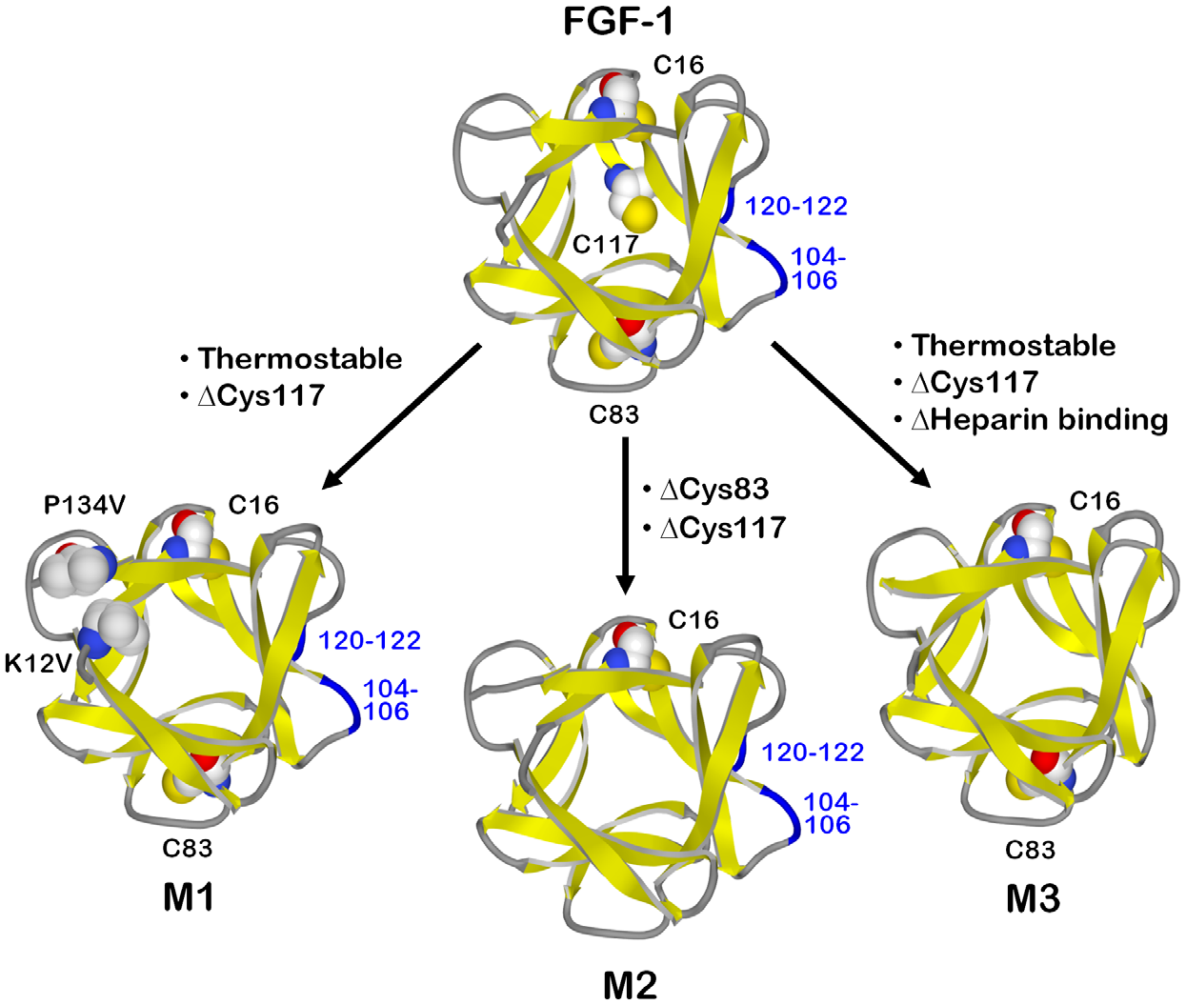
Relationship between WT FGF-1 and mutant proteins. The FGF-1 structure (PDB accession 2AFG [62]) has three buried reactive thiols (Cys residues indicated) and heparin-binding functionality associated with surface loops 104–106 and 120–122 (indicated by blue color). Mutant M1 (based upon PDB accession 2HWM for Lys12Val/Cys117Val mutant [30]) includes stabilizing mutations Lys12Val and Pro134Val (indicated) combined with elimination of one buried thiol (Cys117Val). Mutant M2 (PDB accession 3FGM [31]) combines elimination of two buried thiols (Cys83Thr/Cys117Val) with two fully-buried stabilizing mutations (Leu44Phe/Phe132Trp; not shown) that offset the destabilizing effects of the Cys mutations (such that thermostability of M2 is equivalent to WT FGF-1). Mutant M3 (PDB accession 3O3Q, a Phe108Tyr form of M3 that promotes crystallization [63]) has enhanced thermostability and elimination of one buried thiol (Cys117Val) and is thus similar to M1; however, M3 also has loop deletions (blue turn regions in FGF-1) that effectively eliminate heparin-binding functionality.

**Table 1:** Proteins utilized in the PK study.

Protein	ΔΔG^1^(kJ/mol)	Buried thiols	Heparinbinding site	EC_50_ 2(ng/mL)	Half-life^3^(hr)
FGF-1 plus 3x mass heparin 4,5	∼−20.0	3	Yes	0.48±0.08	
FGF-1 w/o heparin 5,6	0.0	3	Yes	60.0±6.7	1.0
Mutant - M1 6 Lys12Val/Cys117Val/Pro134Val	−17.9	2	Yes	1.8±0.90	N.D.
Mutant - M2 5 Leu44Phe/Cys83Thr/Cys117Val/Phe132Trp	−0.4	1	Yes	0.74±0.19	42.6
Mutant - M3 7 Leu44Phe/Met67Ile/Leu73Val/Val109Leu/Leu111Ile/Cys117Val/Ala103Gly/Arg119Gly/Δ104–106/Δ120–122	−16.1	2	No	0.84±0.22	N.D.

1 A negative value indicates increased thermostability compared to WT FGF-1.

2 Effective concentration for 50% maximum mitogenic activity against 3T3 fibroblasts; a lower value indicates increased effective mitogenic potency.

3 Residual mitogenic activity after cell culture medium incubation at 37°C.

4 [24].

5 [31].

6 [30].

7 [32].

The present report describes a PK study, performed in New Zealand White (NZW) rabbits, of human FGF-1 formulated in the presence and absence of heparin, as well as three mutant forms of FGF-1 (each formulated in the absence of heparin) (Fig. 1). These mutant forms were selected based upon their *in vitro* properties of increased thermostability [30] or reduced number of buried reactive thiols (these two properties cooperatively interact to significantly increase the *in vitro* functional half-life [31]; Table 1). Another mutant is both thermostable and has deletions within the heparin-binding site that result in an order of magnitude reduction in heparin/heparan sulfate proteoglycan (HSPG) affinity [32]. A comparison of the PK parameters for these mutant forms permits an evaluation of the consequences upon the PK profile of heparin in the formulation of FGF-1, as well as the role of HSPG affinity in the overall distribution and elimination kinetics of FGF-1. The results support the hypothesis that the known HSPG sequestration of FGF-1 after intravenous (IV) bolus by liver, kidney and spleen is a key determinant of the PK distribution and elimination profile, and that heparin in the formulation of FGF-1 causes a more endocrine type profile. Enhancing the thermostability and eliminating buried reactive thiols yields a PK profile exhibiting greater efficiency of HSPG sequestration, resulting in less endocrine-type behavior, as well as extended elimination half-life and MRT. The aim of the present study was to determine if PK properties can be manipulated by specific protein mutation; the results show that they can, and that the mutations under study may have significant advantages over the WT FGF-1 protein for therapeutic application.

**Table 2:** Two-compartment IV bolus pharmacokinetic model (Cp = A*e^−αt^ + B*e^−βt^) analysis of wild-type and mutant FGF-1 proteins (n = 3 for each protein).

Protein	FGF+Heparin	FGF w/o Heparin	M1	M2	M3
**Fitted Parameters**					
*A* (µg/ml)	0.975±0.187	0.624±0.0387	0.767±0.314	0.251±0.159	2.41±0.690
*B* (µg/ml)	0.0324±0.00776	0.0329±0.00179	0.0136±0.00063	0.0106±0.00319	0.0905±0.0309
α (min^−1^)	0.0682±0.0049	0.145±0.007	0.333±0.104	0.171±0.057	0.110±0.004
β (min^−1^)	0.00576±0.00044	0.00524±0.00033	0.00277±0.00059	0.00305±0.00067	0.00938±0.00072
**Rate Constants**					
Distr. *t* ^1^/_2_ (min)	10.2±0.7	4.80±0.24	2.26±0.85	4.41±1.69	6.30±0.24
Elim. *t* ^1^/_2_ (min)	121±10	133±8	258±51	234±49	74.0±5.7
*k*_21_ (min^−1^)	0.00777±0.00053	0.0122±0.0005	0.00883±0.00122	0.0105±0.0029	0.0130±0.0006
*k*_10_ (min^−1^)	0.0505±0.0018	0.0619±0.0021	0.107±0.043	0.0514±0.0232	0.0792±0.0052
*k*_12_ (min^−1^)	0.0157±0.0031	0.0757±0.0064	0.221±0.064	0.113±0.038	0.0270±0.0056
**Primary Pharmacokinetic Parameters**					
AUC (min*µg/ml)	20.1±4.48	10.6±0.997	7.27±1.11	4.89±0.900	31.7±9.13
AUMC (min^2^*µg/ml)	1,180±150	1,240±210	1,930±700	1,160±216	1,270±551
V_1_ (ml/kg)	102±22	153±10	154±88	471±217	42.0±10.4
V_2_ (ml/kg)	214±97	944±73	3,421±568	4,747±1,757	84±12
V_2_/V_1_	2.03±0.461	6.19±0.51	25.9±10.4	10.6±1.9	2.08±0.45
V_ss_ (ml/kg)	316±119	1,097±77	3,575±636	5,217±1,970	126±19
MRT (min)	59.9±6.9	116±11	261±62	244±60	39.1±7.1
Cl (ml/min*kg)	5.18±1.32	9.46±0.93	14.0±2.0	20.9±4.0	3.33±0.89

FGF-1 was analyzed with and without 3x mass excess of Heparin; mutant proteins were analyzed in the absence of heparin in each case.

## Materials and Methods

### Mutant FGF-1 Selection

FGF-1 has poor thermostability and three buried reactive thiol groups (i.e., free cysteines) that cooperate to substantially limit the *in vitro* functional half-life (via an irreversible unfolding pathway) [23], [24], [31]. Point mutations that increase thermostability or effectively eliminate buried reactive thiols have been shown to significantly increase the *in vitro* functional half-life, enable full mitogenic potency in the absence of exogenously-added heparin, provide resistance to proteolysis, and reduce disulfide-mediated oligomerization/aggregation [30], [31]. Three mutations were selected to probe the effects of thermostability, buried reactive thiols, and heparin affinity upon the *in vivo* PK profile (Table 1). Mutant M1 is a thermostable mutant whose increase in thermostability approximates the stabilizing effect of bound heparin. M1 includes two point mutations (Lys12Val/Pro134Val) designed to stabilize the N- and C-terminus β-strand interactions, a region of structural weakness in the β-barrel architecture [30] (Fig. 1). M1 also includes a point mutation (Cys117Val) that eliminates one of three buried reactive thiols. Mutant M2 combines four point mutations; two mutations (Cys83Thr/Cys117Val) eliminate two of three buried reactive thiols in the structure (the other being Cys16), and two mutations (Leu44Phe/Phe132Trp) are stabilizing core mutations that offset the destabilizing effects of the Cys mutations [31]. Thus, mutant M2 has thermostability indistinguishable from WT FGF-1, but has eliminated two buried reactive thiols. Mutant M3 combines eight point mutations and six deletion mutations and was developed to increase the overall threefold structural symmetry of FGF-1 [32]–[34]. The heparin binding site is a structural “aneurism” within the overall threefold tertiary structure symmetry characteristic of the β-trefoil fold [32], [35], [36] (Fig. 1). Deletion of the heparin binding site is associated with a marked increase in thermostability but loss of heparin binding functionality (thus supporting the “stability/function tradeoff” hypothesis [37], [38]). Therefore, M3 is similar to M1 in that both have an equivalent increase in thermostability (approximately equal to the effect of heparin addition to WT FGF-1) and both have the same buried reactive thiol eliminated (i.e., Cys117); however, whereas M1 has normal FGF-1 heparin binding site, M3 is deficient in this functionality. Thus, the set of FGF-1±heparin and mutant proteins can effectively probe differential effects of thermostability, buried reactive thiols, and heparin/HSPG interaction upon the *in vivo* PK profile of FGF-1.

**Figure 2:**
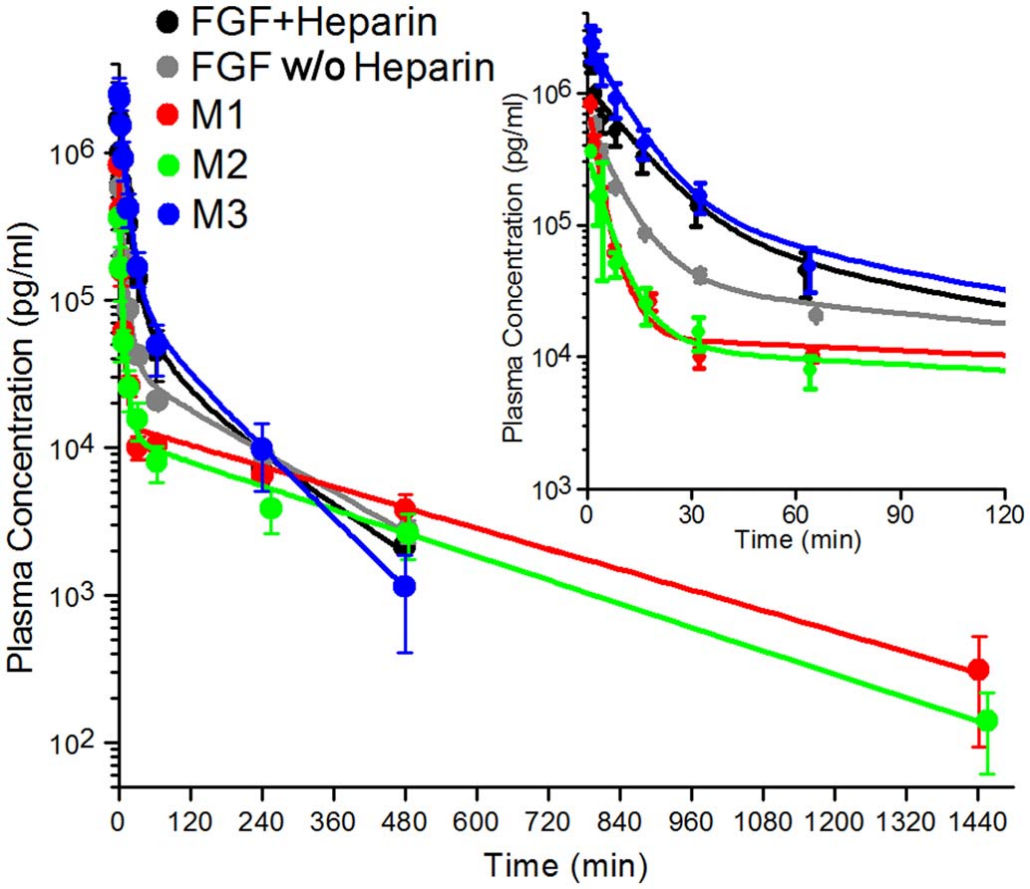
PK profile and fitted two-compartment model for WT and mutant FGF-1 proteins (error bars for n = 3 data sets for each protein). PBX IV bolus yielded no detectable endogenous FGF-1 for any time point (not shown). The inset on upper right shows a close-up of the 0–120 min time period.

### Recombinant Protein Design

The 140 amino acid form of human FGF-1 [39] was utilized throughout this study. Although produced from an *E. coli* expression system, the FGF-1 protein was designed to be as close to the naturally occurring human form as possible. *E. coli* is known to initiate translation with an N-formyl methionine (f-Met) at the N-terminus of expressed proteins. Since f-Met can be recognized by the immune system of eukaryotes as foreign, a cleavable construct was designed to eliminate this prokaryotic modification. Specifically, the 140 amino acid form of FGF-1 was fused downstream of an enterokinase (EK) recognition sequence (Asp_4_Lys) preceded by a flexible 20 amino acid linker (derived from the S-tag sequence of pBAC-3 (EMD Millipore, Billerica MA)) and an N-terminal (His)_6_ tag. The resulting expressed fusion protein utilizes the (His)_6_ tag for efficient purification and is subsequently processed by EK digestion to yield the 140 amino acid form of FGF-1 having a native eukaryotic N-terminus.

**Figure 3:**
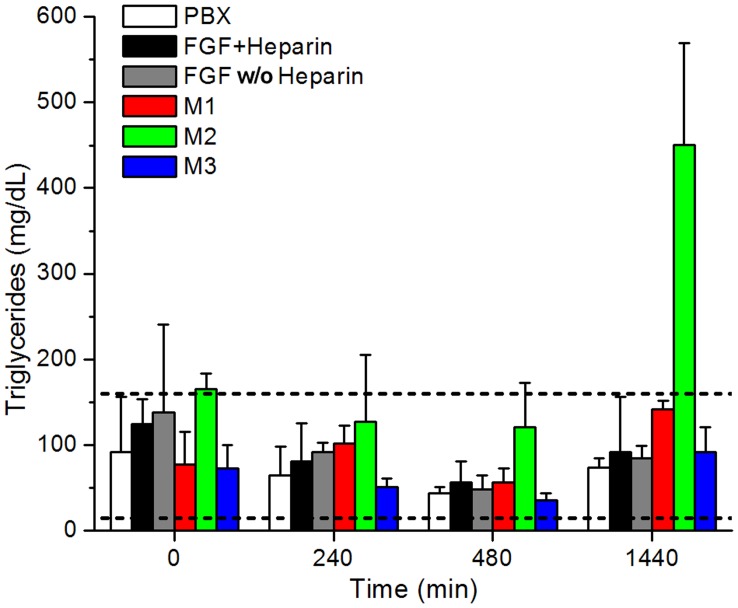
Plasma triglyceride levels for pre-bleed (T = 0) control, 240, 480 and 1440 min samples. PBX control is indicated in white, FGF-1 plus heparin is indicated in black, FGF-1 in the absence of heparin in gray, mutant M1 in red, mutant M2 in green, and mutant M3 in blue. Standard deviation for each protein measurement is indicated by vertical error bar. The normal range for triglyceride levels in NZW rabbits [50] is indicated by the two horizontal dashed lines.

**Figure 4:**
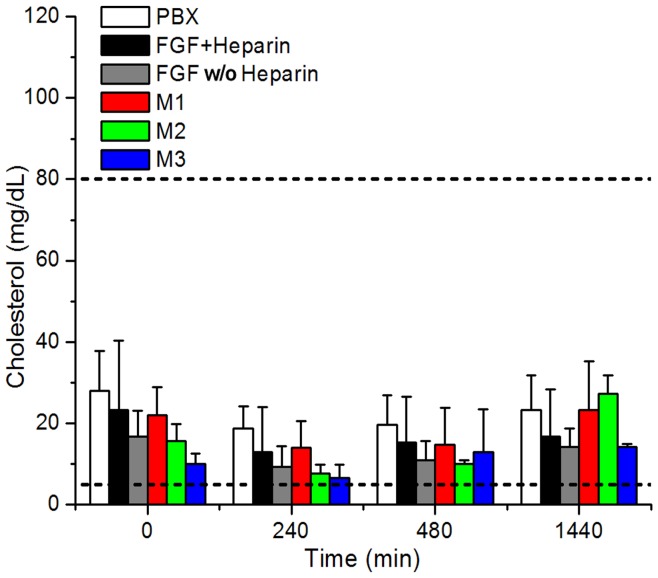
Plasma cholesterol levels for pre-bleed (T = 0) control, 240, 480 and 1440 min samples. Standard deviation for each protein measurement is indicated by vertical error bar. The normal range for cholesterol levels in NZW rabbits [50] is indicated by the two horizontal dashed lines.

**Figure 5:**
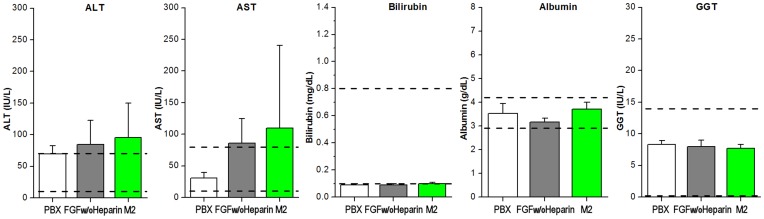
Liver chemistry profile for the 1440 min time point plasma sample from PBX, FGF w/o heparin and M2 mutant protein. The panel includes alanine transaminase (ALT), aspartate transaminase (AST), bilirubin, albumin and γ-glutamyltranspeptidase (GGT). The normal range of these components in the NZW rabbit [50], [64] is indicated by dashed lines (note that the normal low range for GGT is 0.0 IU/L).

### Protein Preparation

Recombinant wild-type (WT) FGF-1 and mutant proteins were expressed from an *E. coli* host after induction with 10 mM isopropyl-β-D-thio-galactoside. The expressed protein was purified utilizing sequential column chromatography on Ni- nitrilotriacetic acid (NTA) affinity resin (Qiagen, Valencia CA) followed by ToyoPearl HW-40S size exclusion chromatography (Tosoh Bioscience, Tokyo). The purified protein was then digested with EK to remove the N-terminal (His)_6_ tag, 20 amino acid linker, and (Asp_4_Lys) EK recognition sequence. A subsequent second Ni-NTA chromatographic step was utilized to remove the released N-terminal peptide (along with any uncleaved fusion protein). Final purification (to ensure monodisperse FGF-1 protein) was performed using HiLoad Superdex 75 size exclusion chromatography (GE Life Sciences, Pittsburgh PA) equilibrated to 50 mM Na_2_PO_4_, 100 mM NaCl, 10 mM (NH_4_)_2_SO_4_, 0.1 mM ethylenediaminetetraacetic acid (EDTA), 5 mM L-Methionine, pH at 6.5 (“PBX” buffer); L-Methionine was included in PBX buffer to limit oxidization of reactive thiols and other potential oxidative degradation. An extinction coefficient of E_280nm_ (0.1%, 1 cm) = 1.26 was utilized for concentration determination of FGF-1 [40], [41]; whereas, E_280nm_ = 1.35 was used for M1, and E_280nm_ = 1.31 was used for both M2 and M3 mutants (based upon the method of Gill and von Hippel [42]). For storage and use in all PK studies, the purified proteins were sterile filtered through a 0.22 micron filter, purged with N_2_, snap frozen in dry ice and stored at -80°C prior to use. The purity of the final proteins was assessed by both Coomassie Brilliant Blue and Silver Stain Plus (BIO-RAD Laboratories, Inc., Hercules CA) stained sodium dodecylsulfate polyacrylamide gel electrophoresis (SDS PAGE). All proteins were prepared in the absence of heparin. Prior to IV bolus, heparin (3x mass; Sigma Chemical, St. Louis MO), or PBX, was added to the WT FGF-1 protein (mutant proteins were diluted with PBX buffer only).

**Figure 6:**
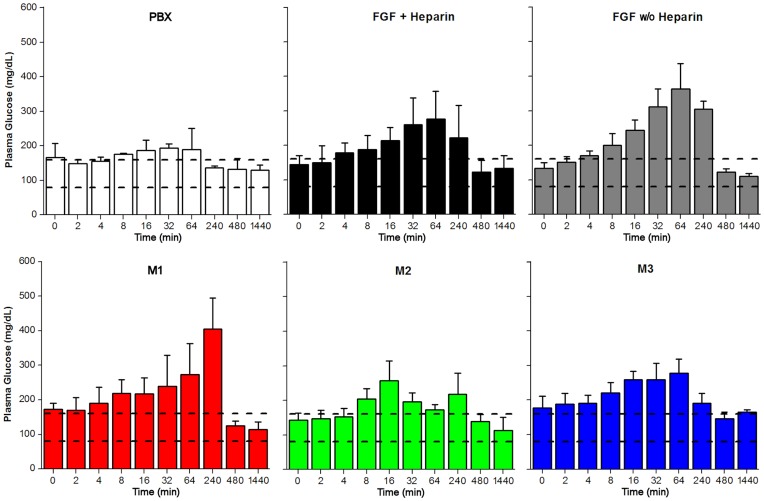
Plasma glucose levels for pre-bleed (T = 0) control and time points from 2 to 1440 min. Standard deviation for each protein measurement are indicated by vertical error bar. The normal range for plasma glucose levels in NZW rabbits [50] is indicated by the two horizontal dashed lines.

### Pharmacokinetics

PK profiles were determined in 6- to 8-month old male NZW rabbits (Robinson Services, Inc., Mocksville NC) weighing 3.3–4.0 kg. Animals were housed in an AAALAC accredited facility in accordance with the Guide for the Care and Use of Laboratory Animals under a 12∶12 hr light:dark cycle, 20°C and 30–70% humidity. Rabbits were chosen for PK study due to fewer amino acid differences between human and rabbit FGF-1 (4 differences) than between human and mouse/rat (5 differences). Additionally, the rabbit hind limb model is a *de facto* standard for the study of induced ischemic disease and treatment by pro-angiogenic growth factors [43]–[46]. Three rabbits per protein (n = 3) were utilized to account for variation in individual response. Rabbits were sedated with 1.0 mg/kg equal dosage of butorphanol tartrate (Fort Dodge, Fort Dodge IA) and acepromazine maleate (VEDCO, St. Joseph MO) through intramuscular injection and with 2% lidocaine jelly (Akorn, Lake Forest IL) applied topically to the ears to facilitate injections and blood collection. A 3.0 ml pre-bleed (i.e., T = 0 min) sample was taken from the right ear artery prior to IV bolus to establish baseline levels of FGF-1 and blood components. 100 µg/kg (330–400 µg per rabbit) of purified WT (+/−3x mass heparin) or mutant FGF-1 protein (w/o heparin in each case) was administered (in 1.0 ml total volume, diluted with PBX) intravenously through the left ear marginal vein. Nominal 3.0 ml bleed volumes were collected using 23 gauge butterfly catheters from the right ear central artery into EDTA coated tubes. Whole blood was centrifuged at 4,000 x g for 30 min and plasma was recovered, snap-frozen in dry ice, and stored at −80°C prior to analysis. Plasma, rather than serum, was collected to avoid potential degradation of FGF-1 by activated coagulation proteases. Nominal bleeding time points were 1, 2, 4, 8, 16, 32, 64 min, 4, 8, and 24 hr post IV bolus; however, due to variations in animal response (shunting, coagulation, artery morphology, *etc*.) some variability in bleed time occurred. Exact times were recorded for each bleed collection and used in the analysis of pharmacokinetic profiles. All procedures were approved by the Florida State University.

**Figure 7:**
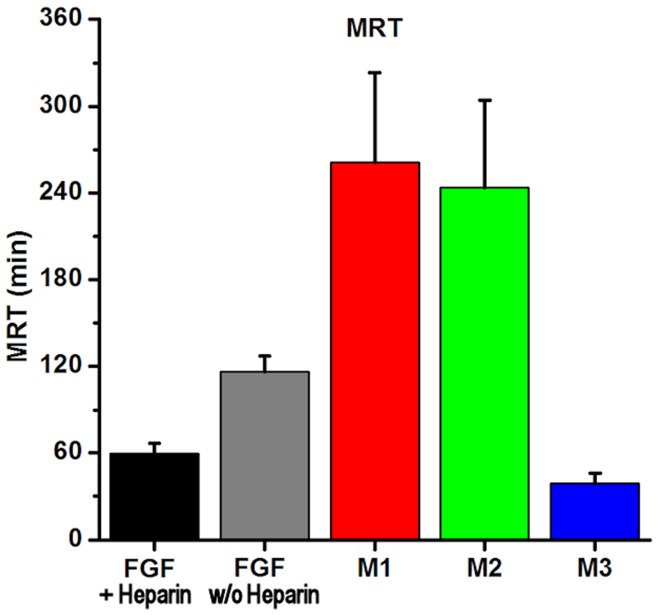
MRT values plotted for all proteins. MRT is reduced for FGF-1+heparin, or for mutant M3 (which has a diminished heparin binding site), showing that heparin sequestration is a prime determinant of MRT.

**Figure 8:**
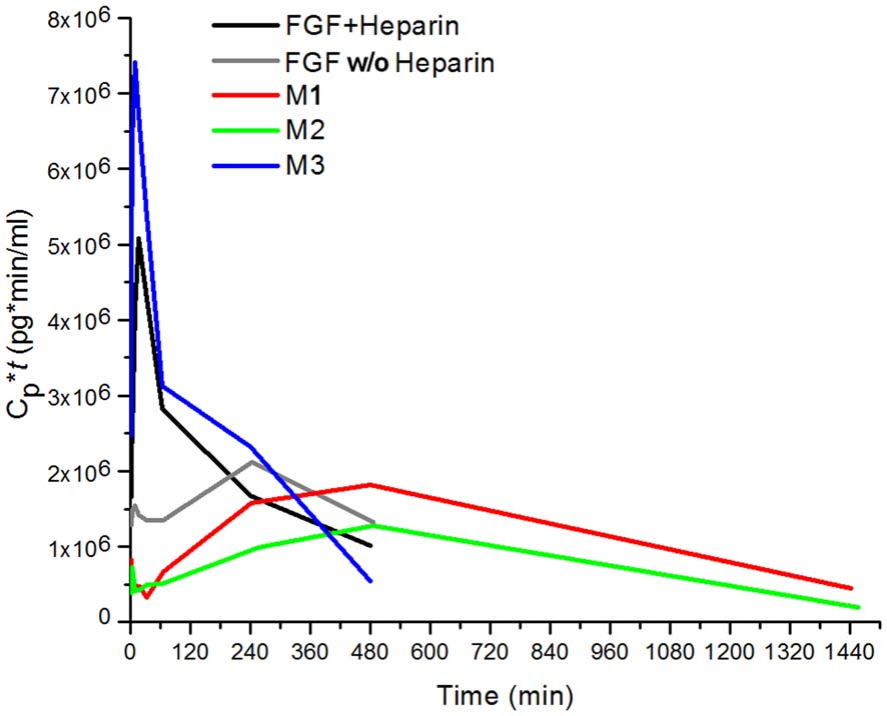
Cp*t curves for all proteins. Efficient distribution of FGF-1 from plasma to HSPG (via the heparin binding site) serves as a storage reservoir for latent redistribution of FGF-1 from HSPG into plasma, extending MRT.

The plasma concentration (Cp) of FGF-1 and mutant proteins was determined using a Quantikine™ human FGF-1 immunoassay (R&D Systems, Inc., Minneapolis MN). This assay has been validated by the manufacturer for plasma samples collected in either EDTA or heparin-coated tubes (i.e., 0.1 mg/ml heparin in plasma); in the present study EDTA coated tubes were selected to avoid unwanted addition of heparin to the plasma samples. In the FGF-1+heparin IV bolus the maximum theoretical heparin concentration in plasma is ∼0.006 mg/ml; thus, this level of heparin, even in undiluted plasma samples, is within the manufacturer’s validated conditions for the enzyme-linked immunosorbent assay (ELISA). The standard curve for each protein was established by a series of 1∶2 dilutions (nominally 2,000 - 32 pg/ml) utilizing the purified WT or mutant FGF-1 proteins. This ELISA assay utilizes a polyclonal capture and detection antibody raised against full-length FGF-1 protein. However, all mutant proteins exhibited varying degree of reduced sensitivity in this ELISA. Postulating that stability effects or residual structure may be responsible, in part, for this reduced sensitivity, 4 M urea/phosphate buffered saline (PBS) was utilized as the assay diluent to promote full denaturation of proteins. This modification to the procedure improved sensitivity of the assay for these mutants, enabling a standard curve that spanned ∼4,000-100 pg/ml for M1, ∼2,000-50 pg/ml for M2, and ∼8,000-150 pg/ml for M3 (with all standard curves generated using 4M urea/PBS). The ELISA absorbance data were quantified using a Synergy H1 Hybrid Multi-Mode Microplate Reader (BioTek Instruments Inc., Winooski VT). The early bleed time points had a much higher FGF-1 concentration than the maximum assayable ELISA concentration; thus, 1∶10, 1∶100, 1∶1,000, and 1∶10,000 dilutions for each plasma sample were initially performed to identify the appropriate dilution for quantitation within the assayable range of the ELISA. Subsequently, appropriately diluted plasma samples were assayed in quadruplicate. The standard curves of protein concentration vs. optical density for each ELISA were fitted using a logistic function (following manufacturer’s instructions), and all plasma samples were quantified by interpolation using appropriate dilutions.

PK profiles for the Cp vs. time data were fit using the *DataFit* non-linear least squares fitting software package (Oakdale Engineering, Oakdale PA) and a two-compartment pharmacokinetic model [47]:
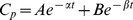
(1)where *A* and α define distribution phase kinetics and *B* and β define elimination phase kinetics, respectively. Macro rate constants were subsequently derived as follows:



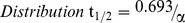
(2)

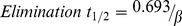
(3)


Micro rate constants *k*_21_ (redistribution rate constant), *k*_10_ (elimination rate constant), and *k*_12_ (distribution rate constant) were derived as follows:
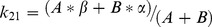
(4)

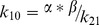
(5)

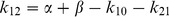
(6)


Primary pharmacokinetic parameters of *Cl* (clearance), *V*_1_ (volume of the central compartment), and *V*_2_ (volume of peripheral compartment) were derived as follows:
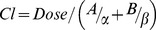
(7)

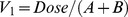
(8)

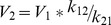
(9)


Other PK parameters, including AUC (area under the curve), *V*_ss_ (volume of distribution under steady state), AUMC (area under the first moment curve), and MRT were derived as follows:
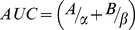
(10)

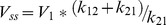
(11)

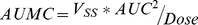
(12)

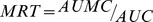
(13)


PK parameters for FGF-1 and mutant proteins were fit independently for each plasma concentration vs. time dataset, and the mean and standard deviation for each n = 3 set were utilized in reporting the derived PK values.

### Plasma Triglyceride, Cholesterol, Liver Chemistry, and Glucose Analyses

Plasma glucose values were measured using a model E4HD109 glucose-dehydrogenase pyrroloquinoline-quinone microfluidics glucose meter (Nipro Diagnostics, Inc., Fort Lauderdale FL). Plasma glucose levels were measured in triplicate for each plasma sample time point. Plasma triglyceride, cholesterol and liver chemistry analyses were performed by the University of Florida Veterinary Diagnostic Laboratories (Gainesville FL).

## Results

### Protein Preparation

The WT FGF-1 and mutant proteins were isolated with >98% purity and homogenous monomeric forms, as judged from non-reduced silver stained SDS PAGE (Fig. S1). The overall yield of purified protein varied from 6–10 mg/L of *E. coli* cell culture, and a 1.0 L culture was sufficient to produce the necessary quantity of each protein for PK study.

### FGF-1 Blood Collection and Pharmacokinetics

Three rabbits presented with either a hyperglycemic or hyperlipidemic condition in their pre-bleed sample and were excluded from the study. One rabbit presented with hypothermia during the study; additionally, several rabbits exhibited torpor subsequent to IV bolus that resulted in a reduced requirement for anesthesia prior to subsequent handling. However, no correlation could be identified between these general observations and a specific protein or PBX control. Plasma samples were visibly unremarkable with the exception of the 24 hr time point for mutant M2. For all rabbits given an IV bolus of mutant M2, the 24 hr time point exhibited a visible opacity (suggesting a possible hyperlipidemic condition). Opaque plasma samples were not observed for any earlier time point or with the 24 hr time point of any other mutant or WT FGF-1 protein. Therefore, additional 48 and 72 hr samples were collected for rabbits treated with mutant M2 for the purpose of visibly evaluating potential hyperlipidemia; subsequently, the cloudiness of the plasma resolved over these later time points. Because of this apparent hyperlipidemia, plasma triglyceride, cholesterol and liver panel assays were performed on all plasma samples.

The FGF-1 ELISA assay can potentially cross-react with rabbit FGF-1; however, blood samples of the PBS control IV bolus, for every time point, and with each rabbit in this control set, yielded undetectable levels of endogenous FGF-1; thus,endogenous levels of rabbit FGF-1 were not an interfering factor. A plot of Log(Cp) vs. time for all data indicated a general bi-exponential decay that was in excellent agreement with a two-compartment model (Fig. S2). Non-linear least squares fitting of the Cp vs. time data demonstrated robust convergence of the fitted parameters regardless of variation of the initial values utilized in the fit. Additionally, deletion of the first or last data point in each set, followed by re-fitting, resulted in <15% change in the refined parameters. Standard error for PK parameters of the n = 3 set for each protein was approximately 15–20% with the exception of the M2 mutant, which yielded higher standard error values. The standard error of the fit to the two-compartment model for individual data sets was on the order of 10–15% (data not shown); thus, the standard error for the fit of the raw data to the model was approximately equivalent to the standard error between data sets. The derived PK constants are given in Table 2 and a summary of the mean and standard deviation values for each protein is provided in Table S1.

Based upon average NZW cardiac output (∼200 ml/min) and blood volume (∼6% mass, or ∼210 ml for a 3.5 kg rabbit) [48], [49], a 1.0 min blood sample time point represents approximately a single pass of an IV bolus through the rabbit circulatory system and is therefore the earliest possible time for which homogenous plasma distribution can be considered. Analysis of the derived pharmacokinetic constants indicated that the 8 hr time point covered 3–5 elimination half-lives for all proteins except M1 and M2. For M1 and M2, data collection to 24 hr was required to cover 3–5 elimination half-lives; thus, PK analyses for all proteins met this requirement for two-compartment PK analysis [47] (Table 2). The 24 hr time point undiluted plasma samples for FGF-1±heparin and M3 mutant proteins were below the detection limit of the ELISA assay; thus, the PK profile for these proteins was analyzed over the 480 min period. The mean PK time points with standard deviation (n = 3) and fitted two-compartment functions for all proteins are provided in Fig. 2. The general PK profile for the various FGF-1 proteins in this study follows a rapid distribution phase, involving ∼99% of the T_0_ bolus concentration, and spanning approximately 20–60 min, prior to establishment of the pseudo-equilibrium elimination phase. The elimination phase for the various FGF-1 proteins could be followed (within the detection limits of the assay) over a period of 8–24 hr and involving another two-orders of magnitude reduction in plasma concentration. A notable variation of distribution and elimination kinetics was observed for the set of FGF-1 proteins, and in each case, there appeared to be a generally inverse correlation between the distribution and elimination kinetics (i.e., a fast distribution was followed by a slow elimination, and vice versa).

### Plasma Lipids and Glucose

Plasma triglyceride levels resided within the normal range (15–160 mg/dL [50]) for NZW rabbits for all 0, 240, 480 and 1440 min (24 hr) time points with the exception of the 24 hr time point for mutant M2 (Fig. 3, Table S2). This time point averaged 451±118 mg/dL, or approximately 3x the normal maximum range, indicating an acute hyperlipidemic condition 24 hr after IV bolus (which agreed with the visible observation of opaque plasma during blood collection). Plasma cholesterol levels fell within the normal range (5–8 mg/dL [50]) for NZW rabbits for all proteins and all time points (Fig. 4, Table S2). A liver chemistry profile was performed on the 24 hr plasma time point of the M2 mutant and compared to PBX control and FGF w/o heparin in an effort to identify any acute liver condition associated with the elevated triglycerides with the M2 24 hr plasma samples. Values for alanine transaminase (ALT), aspartate transaminase (AST), total bilirubin, albumin and γ-glutamyltranspeptidase (GGT) were each within the normal range, although M2 samples exhibited comparatively large standard deviation (especially with ALT and AST values) (Fig. 5, Table S3). EDTA from the blood collection tubes interferes with the alkaline phosphatase (ALP) assay, and so ALP values were not determined.

Plasma glucose levels with the PBX control exhibited no statistically significant variation outside the normally expected range over the 24 hr evaluation period (Fig. 6, Table S4). WT FGF-1 (both in the presence and absence of heparin) induced an apparent acute hyperglycemic condition, peaking at approximately 1 hr post-IV bolus. Mutant M1 exhibited a similar hyperglycemic condition, although the peak glucose level was observed at the later 4 hr time point. Mutant M2 exhibited a less significant hyperglycemia, while the effect upon plasma glucose for mutant M3 was essentially indistinguishable from that of FGF-1+heparin. In all cases, normal plasma glucose levels were restored by the 8 hr time point. Although the highest measured glucose value was 404±91 mg/dL, the danger level for glucose in acute hyperglycemia in rabbits is considered >1,000 mg/dL [51], [52].

The 100 µg/kg dose of FGF-1 or mutant proteins utilized in this PK study is considered to be a “high” dose [8], [9], [53]–[55]. The only significant event observed for the blood components assayed was elevated triglycerides in the 24 hr time point with the M2 mutant protein. This appears to be a transient hyperlipidemia that subsequently resolves over 48–72 hr. It is unclear what the cause of this hyperlipidemia is, since the mutations in the M2 protein are surface inaccessible and do not affect receptor or heparin binding properties, and the overall thermostability of M2 is essentially equivalent to WT FGF-1. Further study is required to understand the cause of the increased triglycerides and whether the effect is absent at lower doses.

## Discussion

Prior PK and imaging studies subsequent to IV bolus of radioiodinated or Technetium labeled FGF-1 in rodents have demonstrated a rapid clearance from plasma with corresponding accumulation in liver, kidney and spleen; and this accumulation correlates with the HSPG distribution in such tissues [53]–[55]. Increased tissue levels of HSPG restrict the distribution of signaling molecules such as FGF-1, can regulate concentration gradients of such signaling molecules, and may play a role in pattern formation in embryogenesis; conversely, decreased levels of HSPG promote long range transport of such signaling molecules [56]. Thus, HSPG binding is postulated to be a key determinant of the PK properties of FGF-1 [53]–[55]. The inclusion of mutants that differentially affect thermostability, number of buried reactive thiols, and heparin affinity, along with FGF-1±heparin, permits an analysis of the differential effects of such parameters upon FGF-1 PK properties. Inclusion of a form of FGF-1 (M3) with a heparin binding site deletion enables a direct experimental test of the role of HSPG in FGF-1 PK.

### Comparison of FGF-1+heparin and FGF-1 w/o heparin

The distribution half-life for FGF-1+heparin is approximately twice as long as FGF-1 w/o heparin(10.2 min vs. 4.8 min, respectively). This result supports HSPG binding as a primary determinant of distribution kinetics for FGF-1 (as heparin occupying the heparin-binding site of FGF-1 must be competed off by HSPG in order for FGF-1 to bind to HSPG). In agreement with this interpretation, V_2_/V_1_ (a measure of the relative distribution of drug over the two compartments, with a larger ratio indicating a greater fraction of drug resides in the peripheral compartment) is three times greater (i.e., 6.2 versus 2.0) for FGF-1 w/o heparin compared to FGF-1+heparin. The MRT of FGF-1 w/o heparin is also twice as great as compared to FGF-1+heparin (i.e., 116 versus 59.9 min, respectively). The elimination half-life is essentially identical for FGF-1±heparin and is consistent with identical PK profile subsequent to distribution (i.e., *k*_12_ is principally determined by binding of FGF-1 to HSPG, which competes with bound heparin; whereas, *k*_21_ is determined by release from HSPG). The increase in MRT for FGF-1 w/o heparin therefore appears due to the increased distribution in the peripheral compartment (i.e., more effective sequestration by HSPG).

### Effect of Enhanced Thermostability; a Comparison of M1 and FGF-1 w/o heparin

The heparin-binding site in FGF-1 is a consequence of the correctly folded structure of FGF-1. FGF-1 has low thermostability and is prone to denaturation [23], [24]; thus, stabilizing the structure might be expected to also confer a more stable heparin binding site with improved efficiency for HSPG binding. The M1 mutant in comparison to FGF-1 w/o heparin exhibits an approximately two-fold shorter distribution half-life (i.e., 2.3 versus 4.8 min, respectively). Notably, while V_1_ for M1 is essentially unaffected in comparison to FGF-1 w/o heparin, V_2_ increases by a factor of four (and V_2_/V_1_ for M1 is 25.9 vs. 6.2 for FGF-1 w/o heparin); furthermore, the MRT for M1 doubles in value in comparison to FGF-1 w/o heparin. These effects with M1 appear due principally to a threefold increase in *k*_12_ distribution constant, consistent with more efficient partitioning of M1 mutant from plasma to HSPG as well as reduction in kinetic constant of redistribution (i.e., *k*_21_). In other words, the increase in thermostability (in addition to the loss of one buried reactive thiol) results in an apparent increased on-rate (*k*_12_) and decreased off-rate (*k*_21_) for HSPG. The elimination kinetic constant (*k*_10_), postulated to be principally determined by kidney excretion, remains essentially unchanged.

### Effect of Removal of Buried Reactive Thiols; a Comparison of M2 and FGF-1 w/o heparin

FGF-1 contains three free cysteines that are chemically reactive and can participate in thiol chemistry (e.g., form mixed thiol adducts), resulting in irreversible unfolding and aggregate formation [31]. Reactivity of such thiols requires accessibility (i.e., protein unfolding) since these positions are buried within the protein core. Thus, there is a cooperative interplay between low thermostability and buried free thiols in the regulation of an irreversible unfolding pathway for FGF-1 [31]. Mutant M2 eliminates two of the three buried reactive thiols in FGF-1 while maintaining equivalent thermostability to FGF-1. Elimination of these two reactive thiols results in a 40-fold longer *in vitro* functional half-life for mutant M2 in comparison to FGF-1 (Table 1). The overall PK rate constants of M2 and FGF-1 w/o heparin are similar, although there is a 1.8x-fold increase in elimination half-life. This appears to be due to a more efficient distribution (*k*_12_), suggesting that chemical reactivity of free cysteine residues in FGF-1 may occur in plasma (with associated reduction of HSPG binding functionality). The M2 data is associated with larger errors than the other proteins; however, as with M1 there is a general increase in V_2_ compared to FGF-1 w/o heparin and consistent with a more efficient overall binding of M2 to HSPG.

### Effect of Diminished Heparin-binding Affinity; a Comparison of M3 and M1

A direct test of the hypothesis that HSPG sequestration is the primary determinant of FGF-1 distribution kinetics could be performed using either an HSPG-deficient organism or a heparin-binding site-deficient FGF-1 (with the former likely being developmentally lethal). Mutant M3 has both enhanced thermostability and substantially diminished HSPG binding affinity (K_d_ for sucrose octasulfate is increased by an order of magnitude [32]). The stability increase of M3 is essentially identical to that of M1 (Table 1), and both have an identical single buried cysteine removed (Cys117); thus, a comparison of M3 and M1 can permit direct evaluation of HSPG affinity upon PK parameters. M3 exhibits a threefold longer distribution half-life and approximately fourfold shorter elimination half-life than M1. These results are consistent with HSPG affinity as a primary determinant of both distribution (*k*_12_) and redistribution (*k*_21_) kinetics of FGF-1. Notably, there is a greater than10x-fold decrease in V_2_/V_1_ for M3 in comparison with M1, principally due to a corresponding decrease in V_2_. In this regard, the V_2_/V_1_ value for M3 is essentially indistinguishable from that of FGF-1+heparin; thus, confirming that bound heparin competes with HSPG for binding of FGF-1 to the peripheral compartment. These results also indicate that the peripheral compartment has a physical interpretation; namely, the HSPG in the vascular walls of kidney, liver and spleen [53]–[55].

### Initial Plasma Concentration

The maximum theoretical equilibrium blood concentration (i.e., assuming no distribution or elimination) in the present study is ∼2.00 µg/ml. A comparison of the extrapolated T_0_ concentration (parameters *A+B* in Table 2) demonstrates essential agreement with this value only for the M3 mutant protein, with varying extent of diminished values for all other proteins. Specifically, the rank order of diminishing (*A+B*) values is M3, followed by FGF-1+heparin (∼1.00 µg/ml), FGF-1 w/o heparin and mutant M1, (both ∼0.700 µg/ml), and mutant M2 (∼0.300 µg/ml). Since the ELISA standard curves utilize the respective mutant proteins, these values do not reflect errors in concentration measurement, rather, the effective removal from plasma of FGF-1 or mutant proteins within a single pass through the circulatory system. Since the initial state describes a non-equilibrium condition (e.g., redistribution rate = 0) this discrepancy between (*A+B*) and theoretical maximum plasma concentration is interpreted as a substantial initial distribution to peripheral compartment during a single circulatory cycle. Even at comparatively high dosages of FGF-1, liver, kidney and spleen have been shown to quantitatively bind FGF-1 from plasma after only a single passage [53]–[55]. Thus, we interpret the rank order of discrepancy between (*A+B*) and maximum theoretical plasma concentration for FGF-1 and mutant proteins as principally based upon differential HSPG binding for these proteins. Thus, M3 with diminished heparin affinity has the greatest observed T_0_ plasma concentration; followed by FGF-1+heparin (where the bound heparin competes with HPSG for FGF-1 binding); then FGF-1 w/o heparin, M1 and M2.

Overall, the present results provide strong experimental support for the hypothesis that HSPG binding serves as the principle physical basis of the peripheral compartment. This, in turn, identifies an important consequence upon PK properties when heparin is utilized in the formulation of FGF-1; specifically, heparin in the formulation will effectively increase the distribution half-life thereby promoting long range transport from the site of delivery and resulting in endocrine-like properties for the introduced FGF-1. The FGF family contains three members (FGF-19, 21 and 23) that lack a functional heparin-binding site and are referred to as “endocrine type” FGF’s [57]. Due to their lack of HSPG binding, these FGFs circulate freely within the blood system, acting at a distance from the site of their synthesis. Thus, the addition of heparin to FGF-1 may promote mitogenic stimulation or angiogenesis distal to the site of delivery. Conversely, eliminating heparin favors local distribution, and may minimize unwanted endocrine type behavior. The endocrine type FGFs can act to reduce levels of plasma glucose and lipids [58]–[61]. Although such effects have not been reported for FGF-1, plasma glucose and lipids were also evaluated as part of this PK study. Daily dosing at 1.0 mg/Kg of FGF-19 over a period of 7 days can reduce plasma glucose levels by 75 mg/dL [58]. In the present study a single IV bolus of 0.1 mg/Kg FGF-1±heparin resulted in an acute *increase* of plasma glucose levels of 150–200 mg/dL (Fig. 6). Thus, the effect of FGF-1 upon plasma glucose level is *opposite* to that observed with the endocrine FGFs, but is of a similar (or greater magnitude) and with a tenfold lower dose (the chronic effects of repeated dosing of FGF-1 upon plasma glucose levels were not evaluated). To our knowledge, this activity of FGF-1 upon plasma glucose levels has not previously been reported. In the case of M2, the observed increase in plasma triglycerides also opposes the effect upon triglyceride levels observed for the endocrine FGFs. The endocrine FGF’s may have uniquely different specificities for the different FGF receptors than FGF-1, and this may determine the observed differential effects upon plasma glucose and triglyceride levels.

An increase in effective HSPG binding is observed for mutants M1 and M2, suggesting that mutational stabilization of the protein, or elimination of buried reactive thiols, can promote more effective HSPG binding. The PK data indicate that these mutational effects contribute to a shorter distribution half-life (i.e., reduced endocrine behavior), as well as an increased elimination half-life (due to slower redistribution kinetics from HSPG-bound protein), resulting overall in a more localized concentration at site of delivery as well as a longer MRT (Table 2, Fig. 7). In effect, the efficient distribution of FGF-1 from plasma to HSPG (via the heparin binding site) stores FGF-1 for later redistribution into plasma (Fig. 8). Thus, the potential benefits of a mutant such as M1 or M2 over wild-type FGF-1 for therapeutic application (e.g., topical application for healing of diabetic ulcers) are multiple, and include the cost benefit in omitting heparin from formulation, safety benefit in elimination of heparin-associated side effects, safety benefit due to reduction in the potential for endocrine type mitogenic activity, therapeutic benefit in increased MRT, and cost and safety benefit in reduction in aggregation, improved storage potential, and potency upon reconstitution.

## Supporting Information

Figure S1 **Recombinant protein purity.** Representative silver stained SDS-PAGE of purified recombinant protein (4 µg FGF-1) resolved in the presence and absence of DTT reducing agent.
(TIF)

Figure S2 **Representative Log(Cp) vs. time data (averaged FGF w/o heparin data set; n = 3) and two-compartment model fit.** The PK data for each protein in the study exhibited a bi-exponential decay that is in excellent agreement with a two-compartment model. The dashed lines indicate the independent distribution and elimination exponential decay functions.
(TIF)

Table S1 **Plasma concentration (Cp) of FGF-1 and mutant proteins and time points utilized in the PK analysis.** The values and standard deviations are for n = 3 in each case.
(DOCX)

Table S2 **Plasma triglyceride (mg/dL) and cholesterol levels (mg/dL).**
(DOCX)

Table S3 **24 hr time point liver chemistry profiles.**
(DOCX)

Table S4 **Plasma glucose levels (mg/dL).**
(DOCX)

## References

[1] Levi-Montalcini R (1964) The Nerve Growth Factor. Annals of the New York Academy of Sciences 118: 149–170. 14258355 10.1111/j.1749-6632.1964.tb33978.x

[2] Hoober JK, Cohen S (1967) Epidermal growth factor. 1. The stimulation of protein and ribonucleic acid synthesis in chick embryo epidermis. Biochimica et Biophysica Acta 138: 347–356. 6048831

[3] Schenkein I, Levy M, Bueker ED, Tokarsky E (1968) Nerve growth factor of very high yield and specific activity. Science 159: 640–643. 5716137 10.1126/science.159.3815.640

[4] Rudland PS, Seifert W, Gospodarowicz D (1974) Growth control in cultured mouse fibroblasts: induction of the pleiotypic and mitogenic responses by a purifed growth factor. Proc Natl Acad Sci U S A 71: 2600–2604. 4527822 10.1073/pnas.71.7.2600 PMC388513

[5] Schumacher B, Stegmann T, Pecher P (1998) The stimulation of neoangiogenesis in the ischemic human heart by the growth factor FGF: first clinical results. J Cardiovasc Surg (Torino) 39: 783–789. 9972900

[6] Stegmann TJ, Hoppert T, Schlurmann W, Gemeinhardt S (2000) First angiogenic treatment of coronary heart disease by FGF-1: long-term results after 3 years. Cardiac and Vascular Regeneration 1: 5–9.

[7] Wagoner L, Merrill W, Jacobs J, Conway G, Boehmer J, et al (2007) Angiogenesis protein therapy with human fibroblast growth factor (FGF-1): results of a phase I open label, dose escalation study in subjects with CAD not eligible for PCI or CABG. Circulation 116 suppl. II443.

[8] Ioli V, Wiens B, Mellin T, Thomas K, Ellis R, et al. (1992) Effect of Topically Applied Acidic Fibroblast Growth Factor (FGF-1) on Healing of Chronic Venous Stasis and Diabetic Ulcers. Abstracts of the 5^th^ Annual Symposium on Advanced Wound Care: 150.

[9] Mellin TN, Mennie RJ, Cashen DE, Ronan JJ, Capparella J, et al (1992) Acidic fibroblast growth factor accelerates dermal wound healing. Growth Factors 7: 1–14. 1380253 10.3109/08977199209023933

[10] Mellin TN, Cashen DE, Ronan JJ, Murphy BS, DiSalvo J, et al (1995) Acidic fibroblast growth factor accelerates dermal wound healing in diabetic mice. Journal of Investigative Dermatology 104: 850–855. 7537778 10.1111/1523-1747.ep12607026

[11] Bing M, Da-Sheng C, Zhao-Fan X, Dao-Feng B, Wei L, et al (2007) Randomized, multicenter, double-blind, and placebo-controlled trial using topical recombinant human acidic fibroblast growth factor for deep partial-thickness burns and skin graft donor site. Wound Repair and Regeneration 15: 795–799. 18028126 10.1111/j.1524-475X.2007.00307.x

[12] Wang W, Lin S, Xiao Y, Huang Y, Tan Y, et al (2008) Acceleration of diabetic wound healing with chitosan-crosslinked collagen sponge containing recombinant human acidic fibroblast growth factor in healing-impaired STZ diabetic rats. Life Sciences 82: 190–204. 18164317 10.1016/j.lfs.2007.11.009

[13] Losordo DW, Vale PR, Symes JF, Dunnington CH, Esakof DD, et al (1998) Gene therapy for myocardial angiogenesis: initial clinical results with direct myocardial injection of phVEGF165 as sole therapy for myocardial ischemia. Circulation 98: 2800–2804. 9860779 10.1161/01.cir.98.25.2800

[14] Rosengart TK, Lee LY, Patel SR, Sanborn TA, Parikh M, et al (1999) Angiogenesis gene therapy: phase I assessment of direct intramyocardial administration of an adenovirus vector expressing VEGF121 cDNA to individuals with clinically significant severe coronary artery disease. Circulation 100: 468–474. 10430759 10.1161/01.cir.100.5.468

[15] Hedman M, Hartikainen J, Syvanne M, Stjernvall J, Hedman A, et al (2003) Safety and feasibility of catheter-based local intracoronary vascular endothelial growth factor gene transfer in the prevention of postangioplasty and in-stent restenosis and in the treatment of chronic myocardial ischemia: phase II results of the Kuopio Angiogenesis Trial (KAT). Circulation 107: 2677–2683. 12742981 10.1161/01.CIR.0000070540.80780.92

[16] Schumacher B, Pecher P, von Specht BU, Stegmann T (1998) Induction of neoangiogenesis in ischemic myocardium by human growth factors: first clincal results of a new treatment of coronary heart disease. Circulation 97: 645–650. 9495299 10.1161/01.cir.97.7.645

[17] Landau C, Jacobs AK, Haudenschild CC (1995) Intrapericardial basic fibroblast growth factor induces myocardial angiogenesis in a rabbit model of chronic ischemia. American Heart Journal 129: 924–931. 7537443 10.1016/0002-8703(95)90113-2

[18] Laham RJ, Rezaee M, Post M, Novicki D, Sellke FW, et al (2000) Intrapericardial delivery of fibroblast growth factor-2 induces neovascularization in a porcine model of chronic myocardial ischemia. J Pharmacol Exp Ther 292: 795–802. 10640320

[19] Pierce GF, Mustoe TA, Altrock BW, Deuel TF, Thomason A (1991) Role of platelet-derived growth factor in wound healing. J Cell Biochem 45: 319–326. 10.1002/jcb.240450403 2045423

[20] Brown RL, Breeden MP, Greenhalgh DG (1994) PDGF and TGF-a act synergistically to improve wound healing in the genetically diabetic mouse. J Surg Res 56: 562–570. 8015312 10.1006/jsre.1994.1090

[21] Jimenez PA, Rampy MA (1999) Keratinocyte growth factor-2 accelerates wound healing in incisional wounds. J Surg Res 81: 238–242. 9927546 10.1006/jsre.1998.5501

[22] Marti GP, Mohebi P, Liu L, Wang J, Miyashita T, et al (2008) KGF-1 for wound healing in animal models. Methods Mol Biol 423: 383–391. 18370216 10.1007/978-1-59745-194-9_30

[23] Blaber SI, Culajay JF, Khurana A, Blaber M (1999) Reversible thermal denaturation of human FGF-1 induced by low concentrations of guanidine hydrochloride. Biophysical Journal 77: 470–477. 10388772 10.1016/S0006-3495(99)76904-3 PMC1300344

[24] Copeland RA, Ji H, Halfpenny AJ, Williams RW, Thompson KC, et al (1991) The structure of human acidic fibroblast growth factor and its interaction with heparin. Archives of Biochemistry and Biophysics 289: 53–61. 1716876 10.1016/0003-9861(91)90441-k

[25] Alsenaidy MA, Wang T, Kim JH, Joshi SB, Lee J, et al (2012) An empirical phase diagram approach to investigate conformational stability of “second-generation” functional mutants of acidic fibroblast growth factor (FGF-1). Protein Sci 21: 418–432. 22113934 10.1002/pro.2008 PMC3375442

[26] Seitz CS, Brocker EB, Trautman A (2008) Management of allergy to heparins in postoperative care: subcutaneous allergy and intravenous tolerance. Dermatology 14: 4. 19061586

[27] Prechel M, Walenga JM (2012) Heparin-induced thrombocytopenia: an update. Seminars in Thrombostasis and Hemostasis 38: 483–496. 10.1055/s-0032-1306432 22399304

[28] Matuszewska B, Keogan M, Fisher DM, Soper KA, Hoe C-M, et al (1994) Acidic fibroblast growth factor: evaluation of topical formulations in a diabetic mouse wound healing model. Pharmaceutical Research 11: 65–71. 7511240 10.1023/a:1018993610801

[29] Kurtzman AL, Govindarajan S, Vahle K, Jones JT, Heinrichs v, et al (2001) Advances in directed protein evolution by recursive genetic recombination: applications to therapeutic proteins. Curr Opin Biotech 12: 361–370. 11551464 10.1016/s0958-1669(00)00228-7

[30] Dubey VK, Lee J, Somasundaram T, Blaber S, Blaber M (2007) Spackling the crack: stabilizing human fibroblast growth factor-1 by targeting the N and C terminus beta-strand interactions. J Mol Biol 371: 256–268. 17570396 10.1016/j.jmb.2007.05.065

[31] Lee J, Blaber M (2009) The interaction between thermostability and buried free cysteines in regulating the functional half-life of fibroblast growth factor-1. Journal of Molecular Biology 393: 113–127. 19695265 10.1016/j.jmb.2009.08.026

[32] Brych SR, Dubey VK, Bienkiewicz E, Lee J, Logan TM, et al (2004) Symmetric primary and tertiary structure mutations within a symmetric superfold: a solution, not a constraint, to achieve a foldable polypeptide. J Mol Biol 344: 769–780. 15533444 10.1016/j.jmb.2004.09.060

[33] Brych SR, Blaber SI, Logan TM, Blaber M (2001) Structure and stability effects of mutations designed to increase the primary sequence symmetry within the core region of a β-trefoil. Protein Science 10: 2587–2599. 11714927 10.1110/ps.ps.34701 PMC2374030

[34] Brych SR, Kim J, Logan TM, Blaber M (2003) Accommodation of a highly symmetric core within a symmetric protein superfold. Protein Science 12: 2704–2718. 14627732 10.1110/ps.03374903 PMC2366980

[35] McLachlan AD (1979) Three-fold structural pattern in the soybean trypsin inhibitor (Kunitz). Journal of Molecular Biology 133: 557–563. 537058 10.1016/0022-2836(79)90408-x

[36] Murzin AG, Lesk AM, Chothia C (1992) β-Trefoil fold. Patterns of structure and sequence in the kunitz inhibitors interleukins-1β and 1α and fibroblast growth factors. Journal of Molecular Biology 223: 531–543. 1738162 10.1016/0022-2836(92)90668-a

[37] Beadle BM, Shoichet BK (2002) Structural basis of stability - function tradeoffs in enzymes. Journal of Molecular Biology 321: 285–296. 12144785 10.1016/s0022-2836(02)00599-5

[38] Tokuriki N, Stricher F, Serrano L, Tawfik DS (2008) How protein stability and new functions trade off. PLoS Comput Biol 4: e1000002. 18463696 10.1371/journal.pcbi.1000002 PMC2265470

[39] Gimenez-Gallego G, Conn G, Hatcher VB, Thomas KA (1986) The complete amino acid sequence of human brain-derived acidic fibroblast growth factor. Biochemical and Biophysical Research Communications 128: 611–617. 10.1016/s0006-291x(86)80540-x 3527167

[40] Tsai PK, Volkin DB, Dabora JM, Thompson KC, Bruner MW, et al (1993) Formulation design of acidic fibroblast growth factor. Pharmaceutical Research 10: 649–659. 7686672 10.1023/a:1018939228201

[41] Zazo M, Lozano RM, Ortega S, Varela J, Diaz-Orejas R, et al (1992) High-level synthesis in *Escherichia coli* of a shortened and full-length human acidic fibroblast growth factor and purification in a form stable in aqueous solutions. Gene 113: 231–238. 1374047 10.1016/0378-1119(92)90400-j

[42] Gill SC, von Hippel PH (1989) Calculation of protein extinction coefficients from amino acid sequence data. Anal Biochem 182: 319–326. 2610349 10.1016/0003-2697(89)90602-7

[43] Little RA (1969) Local and general responses to injury in the newborn rabbit. Postgrad Med J 45: 559–561. 5343587 10.1136/pgmj.45.526.559 PMC2466911

[44] Baffour R, Berman J, Garb JL, Rhee SW, Kaufman J, et al (1992) Enhanced angiogenesis and growth of collaterals by in vivo administration of recombinant basic fibroblast growth factor in a rabbit model of acute lower limb ischemia: dose-response effect of basic fibroblast growth factor. Journal of Vascular Surgery 16: 181–192. 1379646

[45] Pu L-Q, Sniderman AD, Brassard R, Lachapelle KJ, Graham AM, et al (1993) Enhanced revascularization of the ischemic limb by angiogenic therapy. Circulation 88: 208–215. 8319335 10.1161/01.cir.88.1.208

[46] Pu L-Q, Jackson S, Lachapelle KJ, Arekat Z, Graham AM, et al (1994) A persistent hindlimb ischemia model in rabbit. Journal of Investigative Surgery 7: 49–60. 8003465 10.3109/08941939409018282

[47] Rosenbaum S (2011) Basic pharmacokinetics and pharmacodynamics - an integrated textbook and computer simulations. Hoboken: John Wiley & Sons, Inc.

[48] Hgashiyama A, Watkins MW, Chen Z, LeWinter MM (1995) Effects of EMD 57033 on Contraction and Relaxation in Isolated Rabbit Hearts. Circulation 92: 3094–3104. 7586281 10.1161/01.cir.92.10.3094

[49] Preckel B, Schlack W, Heibel T, Rutten H (2002) Xenon produces minimal haemodynamic effects in rabbits with chronically compromised left ventricular function. British Journal of Anaesthesia 88: 264–269. 11878658 10.1093/bja/88.2.264

[50] Evans GO, editor (2009) Animal clinical chemistry - a practical handbook for toxicologists and biomedical researchers. Boca Raton: Taylor & Francis Group LLC.

[51] Thrall MA, Baker DC, Campbell TW, DeNicola DB, Fettman MJ, et al. (2004) Veterinary hematology and clinical chemistry. Hoboken: Wiley Blackwell Publishing.

[52] Willard MD, Tvedten H (2007) Small animal clinical diagnosis by laboratory methods. Philadelphia: W.B. Saunders Co.

[53] Rosengart TK, Kuperschmid JP, Maciag T, Clark RE (1989) Pharmacokinetics and distribution of heparin-binding growth factor-1 (endothelial cell growth factor) in the rat. Circulation Research 64: 227–234. 2463884 10.1161/01.res.64.2.227

[54] Hondermarck H, Courty J, Boilly B, Thomas D (1990) Distribution of intravenously administered acidic and basic fibroblast growth factors in mouse. Experientia 46: 973–974. 1698658 10.1007/BF01939392

[55] Zinn KR, Kelpke S, Chaudhuri TR, Sugg T, Mountz JM, et al (2000) Imaging Tc-99m-labeled FGF-1 targeting in rats. Nuclear Medicine & Biology 27: 407–414. 10938477 10.1016/s0969-8051(00)00090-1

[56] Hacker U, Nybakken K, Perrimon N (2005) Heparan sulphate proteoglycans: the sweet side of development. Nature Rev 6: 530–541. 10.1038/nrm1681 16072037

[57] Beenken A, Mohammadi M (2009) The FGF family: biology, pathophysiology and therapy. Nature Rev 8: 235–253. 10.1038/nrd2792 PMC3684054 19247306

[58] Fu L, John LM, Adams SH, Yu XX, Tomlinson E, et al (2004) Fibroblast growth factor 19 increases metabolic rate and reverses dietary and leptin-deficient diabetes. Endocrinology 145: 2594–2603. 14976145 10.1210/en.2003-1671

[59] Kharitonenkov A, Shiyanova TL, Koester A, Ford AM, Micanovic R, et al (2005) FGF-21 as a novel metabolic regulator. J Clin Invest 115: 1627–1635. 15902306 10.1172/JCI23606 PMC1088017

[60] Ryden M (2009) Fibroblast growth factor 21: an overview from a clinical perspective. Cell Mol Life Sci 66: 2067–2073. 19277467 10.1007/s00018-009-0003-9 PMC11115664

[61] Reiche M, Bachmann A, Lossner U, Bluher M, Stumvoll M, et al (2010) Fibroblast growth factor 19 serum levels: relation to renal function and metabolic parameters. Horm Metab Res 42: 178–181. 20013647 10.1055/s-0029-1243249

[62] Blaber M, DiSalvo J, Thomas KA (1996) X-ray crystal structure of human acidic fibroblast growth factor. Biochemistry 35: 2086–2094. 8652550 10.1021/bi9521755

[63] Lee J, Blaber SI, Dubey VK, Blaber M (2011) A polypeptide “building block” for the ß-trefoil fold identified by “top-down symmetric deconstruction”. J Mol Biol 407: 744–763. 21315087 10.1016/j.jmb.2011.02.002

[64] Hewitt CD, Innes DJ, Savory J, Wills MR (1989) Normal biochemical and hematological values in New Zealand White rabbits. Clinical Chemistry 35: 1777–1779. 2758652

