# Supplementary figures and images for: Pharmacokinetic Properties of 2nd-Generation Fibroblast Growth Factor-1 Mutants for Therapeutic Application
Source: PLoS One. 2012 Nov 1;7(11):e48210. doi: 10.1371/journal.pone.0048210 (PMC3486806; doi:10.1371/journal.pone.0048210)

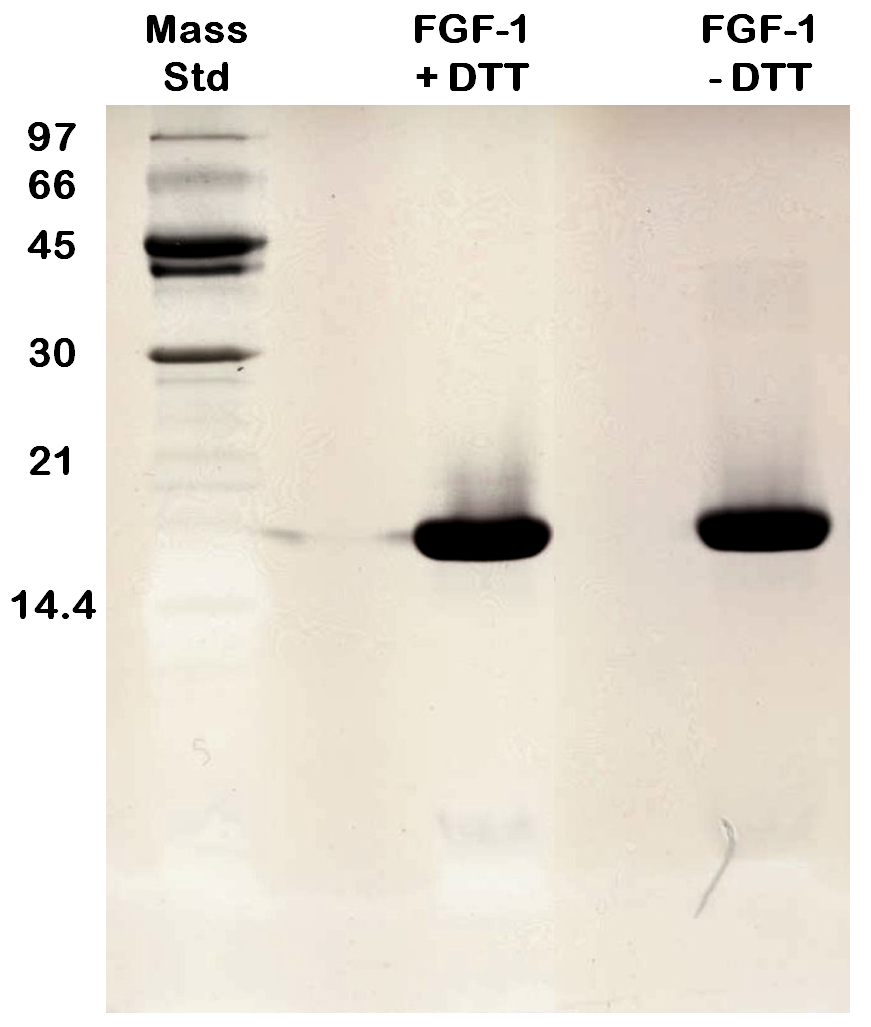

Supplement: Figure S1 — Recombinant protein purity. Representative silver stained SDS-PAGE of purified recombinant protein (4 µg FGF-1) resolved in the presence and absence of DTT reducing agent. (TIF) [file pone.0048210.s001.tif]

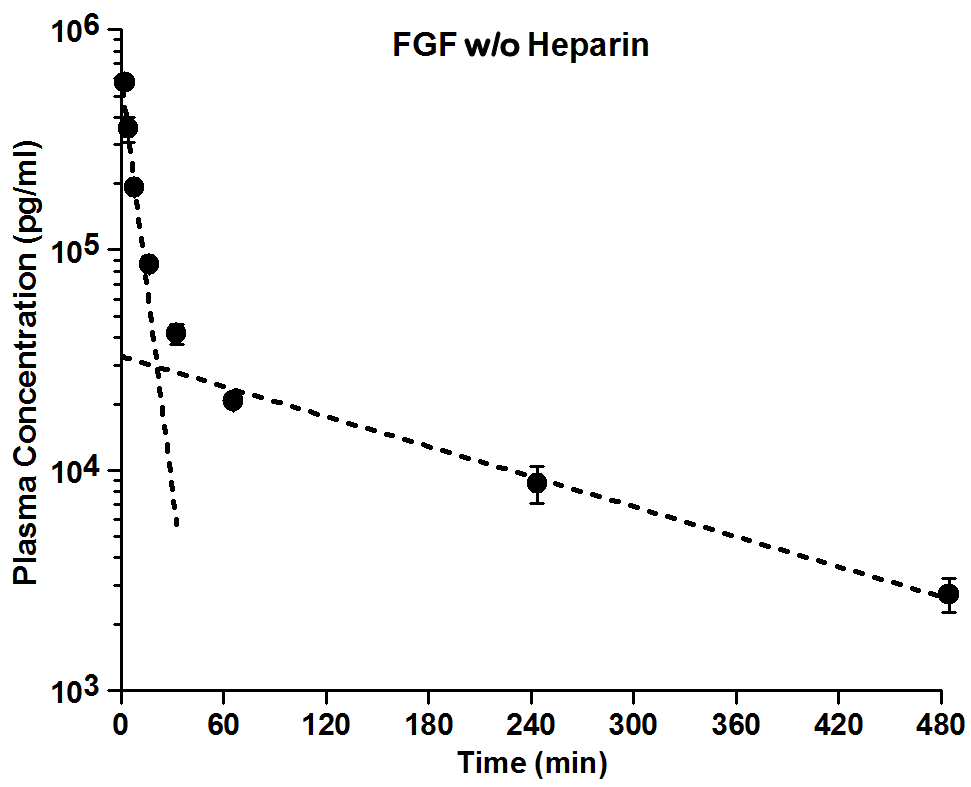

Supplement: Figure S2 — Representative Log(Cp) vs. time data (averaged FGF w/o heparin data set; n = 3) and two-compartment model fit. The PK data for each protein in the study exhibited a bi-exponential decay that is in excellent agreement with a two-compartment model. The dashed lines indicate the independent distribution and elimination exponential decay functions. (TIF) [file pone.0048210.s002.tif]
