# Supplementary material for: Pharmacokinetic Properties of 2nd-Generation Fibroblast Growth Factor-1 Mutants for Therapeutic Application
Source: PLoS One. 2012 Nov 1;7(11):e48210. doi: 10.1371/journal.pone.0048210 (PMC3486806; doi:10.1371/journal.pone.0048210)
Supplement: Table S1 — Plasma concentration (Cp) of FGF-1 and mutant proteins and time points utilized in the PK analysis. The values and standard deviations are for n = 3 in each case. (DOCX) [file pone.0048210.s003.docx]

| Table S1. Plasma concentration (Cp) of FGF-1 and mutant proteins and time points utilized in the PK analysis. The values and standard deviations are for n=3 in each case. | | | | | | | | | | | | | | |
| --- | --- | --- | --- | --- | --- | --- | --- | --- | --- | --- | --- | --- | --- | --- |
| **FGF+Heparin** | | | **FGF w/o Heparin** | | | **Mutant M1** | | | **Mutant M2** | | | **Mutant M3** | | |
| Time (min) | Conc  (μg/ml) | σ  (μg/ml) | Time (min) | Conc  (μg/ml) | σ  (μg/ml) | Time (min) | Conc  (μg/ml) | σ  (μg/ml) | Time (min) | Conc  (μg/ml) | σ  (μg/ml) | Time (min) | Conc  (μg/ml) | σ  (μg/ml) |
| 1.00 | 1.66 | 0.247 | . |  |  | 1.00 | 0.819 | 0.0284 | 1.08 | 0.359 |  | 1.00 | 2.49 | 0.729 |
| 2.25 | 0.991 | 0.0393 | 2.23 | 0.578 | 0.0383 | 2.00 | 0.406 | 0.0714 | 2.86 | 0.164 | 0.0654 | 2.00 | 2.32 | 0.625 |
| 4.00 | 0.626 | 0.130 | 4.19 | 0.355 | 0.0468 | 4.08 | 0.157 | 0.0330 | 4.33 | 0.166 | 0.128 | 4.19 | 1.52 | 0.402 |
| 8.00 | 0.518 | 0.124 | 8.00 | 0.193 | 0.0111 | 7.94 | 0.0608 | 0.00892 | 8.08 | 0.0512 | 0.0108 | 8.17 | 0.907 | 0.264 |
| 15.7 | 0.323 | 0.00796 | 16.50 | 0.0863 | 0.00445 | 18.22 | 0.0262 | 0.00413 | 16.81 | 0.0253 | 0.00774 | 16.17 | 0.416 | 0.104 |
| 31.3 | 0.139 | 0.00420 | 32.44 | 0.0417 | 0.00436 | 32.25 | 0.0100 | 0.00181 | 32.00 | 0.0155 | 0.00438 | 32.33 | 0.166 | 0.0434 |
| 62.7 | 0.0451 | 0.00169 | 65.83 | 0.0206 | 0.00100 | 64.86 | 0.0103 | 0.00113 | 64.00 | 0.00798 | 0.00222 | 64.00 | 0.0490 | 0.0184 |
| 240 | 0.00700 | 0.00130 | 243.33 | 0.00871 | 0.00166 | 241.83 | 0.00652 | 0.00063 | 255.81 | 0.00386 | 0.00123 | 240.33 | 0.00970 | 0.00462 |
| 480 | 0.00213 | 0.00006 | 484.33 | 0.00273 | 0.00048 | 480.33 | 0.00380 | 0.00099 | 485.33 | 0.00263 | 0.00088 | 479.83 | 0.00114 | 0.00073 |
|  |  |  |  |  |  | 1442.33 | 0.00031 | 0.00022 | 1455.33 | 0.00014 | 0.00008 |  |  |  |
