# Supplementary material for: Pharmacokinetic Properties of 2nd-Generation Fibroblast Growth Factor-1 Mutants for Therapeutic Application
Source: PLoS One. 2012 Nov 1;7(11):e48210. doi: 10.1371/journal.pone.0048210 (PMC3486806; doi:10.1371/journal.pone.0048210)
Supplement: Table S2 — Plasma triglyceride (mg/dL) and cholesterol levels (mg/dL). (DOCX) [file pone.0048210.s004.docx]

| Table S2. Plasma triglyceride (mg/dL) and cholesterol levels (mg/dL). | | | | | | |
| --- | --- | --- | --- | --- | --- | --- |
| **Triglycerides** | | | | | | |
| **Time**  **(hr)** | **PBX** | **FGF +Heparin** | **FGF**  **w/o Heparin** | **M1** | **M2** | **M3** |
| 0 | 91.7±64.5 | 124.7±28.9 | 138±102.5 | 77.7±37.8 | 166±17.8 | 73.0±26.9 |
| 4 | 64.3±33.7 | 80.7±45.3 | 91.7±11.6 | 102.3±20.4 | 128±77.7 | 50.7±10.7 |
| 8 | 43.7±7.5 | 56.7±24.1 | 48.7±16.5 | 56.3±16.2 | 121±52.1 | 35.7±7.8 |
| 24 | 74.3±10.6 | 92.0±64.2 | 84.3±14.7 | 141.7±9.7 | 451±118 | 92.0±29.1 |
| **Cholesterol** | | | | | | |
| 0 | 28.0±9.8 | 23.3±17.0 | 16.7±6.4 | 22±7.0 | 15.7±4.2 | 10.0±2.6 |
| 4 | 18.7±5.5 | 13.0±11.1 | 9.3±5.1 | 14±6.6 | 7.7±2.1 | 6.7±3.1 |
| 8 | 19.7±7.2 | 15.3±11.2 | 11.0±4.6 | 14.7±9.1 | 10.0±1.0 | 13.0±10.4 |
| 24 | 23.3±8.5 | 16.7±11.7 | 14.3±4.5 | 23.3±11.9 | 27.3±4.5 | 14.3±0.6 |
