# Supplementary material for: Pharmacokinetic Properties of 2nd-Generation Fibroblast Growth Factor-1 Mutants for Therapeutic Application
Source: PLoS One. 2012 Nov 1;7(11):e48210. doi: 10.1371/journal.pone.0048210 (PMC3486806; doi:10.1371/journal.pone.0048210)
Supplement: Table S3 — 24 hr time point liver chemistry profiles. (DOCX) [file pone.0048210.s005.docx]

| Table S3. 24 hr time point liver chemistry profile | | | | | |
| --- | --- | --- | --- | --- | --- |
|  | **ALT**  **(IU/L)** | **AST**  **(IU/L)** | **Bilirubin**  **(mg/dL)** | **Albumin**  **(g/dL)** | **GGT**  **(IU/L)** |
| PBX | 69.7±12.6 | 30.7±9.2 | 0.09±0.01 | 3.53±0.42 | 8.3±0.6 |
| FGF w/o Heparin | 84.7±37.9 | 86.0±38.5 | 0.09±0.01 | 3.17±0.15 | 8.0±1.0 |
| M2 | 95.7±54.1 | 110±131 | 0.09±0.01 | 3.67±0.31 | 7.7±0.6 |
