# Supplementary material for: Pharmacokinetic Properties of 2nd-Generation Fibroblast Growth Factor-1 Mutants for Therapeutic Application
Source: PLoS One. 2012 Nov 1;7(11):e48210. doi: 10.1371/journal.pone.0048210 (PMC3486806; doi:10.1371/journal.pone.0048210)
Supplement: Table S4 — Plasma glucose levels (mg/dL). (DOCX) [file pone.0048210.s006.docx]

| Table S4. Plasma glucose levels (mg/dL) | | | | | | |
| --- | --- | --- | --- | --- | --- | --- |
| **Time (min)** | **PBX** | **FGF +Heparin** | **FGF**  **w/o Heparin** | **M1** | **M2** | **M3** |
| 0 | 167±41 | 144±25 | 133±17 | 172±19 | 142±21 | 177±35 |
| 2 | 149±11 | 149±49 | 151±17 | 169±37 | 146±25 | 188±32 |
| 4 | 156±12 | 179±27 | 170±14 | 190±46 | 152±25 | 191±23 |
| 8 | 175±4 | 188±41 | 200±34 | 219±40 | 203±31 | 221±30 |
| 16 | 186±31 | 213±38 | 243±30 | 217±46 | 256±58 | 260±24 |
| 32 | 194±11 | 260±77 | 311±52 | 239±90 | 196±25 | 260±46 |
| 64 | 190±60 | 276±81 | 363±74 | 273±90 | 173±14 | 278±40 |
| 240 | 137±4 | 221±94 | 305±23 | 404±91 | 218±60 | 191±29 |
| 480 | 133±31 | 122±34 | 122±9 | 124±15 | 139±19 | 146±20 |
| 1440 | 129±16 | 133±37 | 110±8 | 114±22 | 112±39 | 166±6 |
